# Long term outcomes of intracarotid arterial transfusion of circulatory-derived autologous CD34 + cells for acute ischemic stroke patients—A randomized, open-label, controlled phase II clinical trial

**DOI:** 10.1186/s13287-024-04021-7

**Published:** 2024-11-20

**Authors:** Hung-Sheng Lin, Pei-Hsun Sung, Shu-Hua Huang, Wei-Che Lin, John Y. Chiang, Ming-Chun Ma, Yi-Ling Chen, Kuan-Hung Chen, Fan-Yen Lee, Sheung-Fat Ko, Hon-Kan Yip

**Affiliations:** 1 Department of Neurology, Cognition and Aging Center, Kaohsiung Chang Gung Memorial Hospital and Chang Gung University College of Medicine, Kaohsiung, 83301 Taiwan; 2 Division of Cardiology, Department of Internal Medicine, Kaohsiung Chang Gung Memorial Hospital and Chang Gung University College of Medicine, 123, Dapi Road, Niaosung Dist., Kaohsiung City, 83301 Taiwan; 3 Institute for Translational Research in Biomedicine, Kaohsiung Chang Gung Memorial Hospital, Kaohsiung, 83301 Taiwan; 4 Center for Shockwave Medicine and Tissue Engineering, Kaohsiung Chang Gung Memorial Hospital, Kaohsiung, 83301 Taiwan; 5 Department of Nuclear Medicine, Kaohsiung Chang Gung Memorial Hospital and, Chang Gung University College of Medicine, Kaohsiung, 83301 Taiwan; 6 Department of Diagnostic Radiology, Kaohsiung Chang Gung Memorial Hospital, and Chang Gung University College of Medicine, Kaohsiung, 83301 Taiwan; 7 Department of Computer Science and Engineering, National Sun Yat-Sen University, Kaohsiung, 804201 Taiwan; 8 Department of Healthcare Administration and Medical Informatics, Kaohsiung Medical University, Kaohsiung, 80708 Taiwan; 9 Department of Internal Medicine, Division of Hema-Oncology, Kaohsiung Chang Gung Memorial Hospital and Chang Gung University College of Medicine, Kaohsiung, 83301 Taiwan; 10 Department of Anesthesiology, Kaohsiung Chang Gung Memorial Hospital and, Chang Gung University College of Medicine, Kaohsiung, 83301 Taiwan; 11 Department of Surgery, Division of Cardiovascular Surgery, Kaohsiung Chang Gung Memorial Hospital and Chang Gung University College of Medicine, Kaohsiung, 83301 Taiwan; 12 Department of Radiology, Kaohsiung Chang Gung Memorial Hospital and Chang Gung University College of Medicine, Kaohsiung, 83301 Taiwan; 13 Department of Medical Research, China Medical University Hospital, China Medical University, Taichung, 40402 Taiwan

**Keywords:** Endothelial progenitor cells, Acute ischemic stroke, Intracarotid transfusion of CD34 + cells, Angiogenesis, Neurological outcomes

## Abstract

**Background:**

This phase II randomized controlled trial tested whether the intracarotid arterial administration (ICAA) of autologous CD34 + cells to patients within 14 ± 7 days after acute ischemic stroke (IS) could be safe and further improve short- and long-term outcomes.

**Methods:**

Between January 2018 and March 2022, 28 consecutive patients were equally randomly allocated to the cell-treated group (CD34 + cells/3.0 × 10^7^/patient) or the control group (receiving optimal medical therapy). CD34 + cells were transfused into the ipsilateral brain infarct zone of cell-treated patients via the ICAA in the catheterization room.

**Results:**

The results demonstrated 100% safety and success rates for the procedure, and no long-term tumorigenesis was observed in cell-treated patients. In cell-treated patients, the angiogenesis capacity of circulating endothelial progenitor cells (EPCs)/Matrigel was significantly greater after treatment than before treatment with granulocyte colony-stimulating factor (all *p* < 0.001). Blood samples from the right internal jugular vein of the cell-treated patients presented significantly greater levels of the stromal cell-derived factor 1α/EPC at 5, 10 and 30 min compared with 0 min (all *p* < 0.005). The National Institute of Health Stroke Scale scores were similar upon presentation, but a greater response was observed by Days 30 and 90 in the cell-treated group than in the control group. Tc-99 m brain perfusion was significantly greater at 180 days in the cell-treated group than in the control group (*p* = 0.046). The combined long-term end points (defined as death/recurrent stroke/or severe disability) were notably lower in the control group compared with the cell-treated group (14.3% vs. 50.0%, *p* = 0.103).

**Conclusion:**

Intracarotid transfusion of autologous CD34 + cells is safe and might improve long-term outcomes in patients with acute IS.

*Trial registration* ISRCTN, ISRCTN15677760. Registered 23 April 2018- Retrospectively registered, https://doi.org/10.1186/ISRCTN15677760

## Introduction

Stroke, a growing epidemic, remains the 2nd leading cause of death and the 3rd leading cause of combined death and disability worldwide [1–4]. Although various etiologies are implicated in stroke, atheroembolic stroke remains the most common cause of ischemic stroke (IS) worldwide [5–9]. After the first IS occurs, there is clearly an increased risk for recurrent IS [10–12]. Additionally, previous clinical studies have shown that patients with a recent transient ischemic attack (TIA) or IS with severe stenosis (defined as 70–90% stenosis of the diameter of a major intracranial artery) are at particularly high risk of recurrent stroke in the territory of the stenotic arterial supplied area (estimated to be approximately 23% at 1 year) despite treatment with aspirin and standard management of vascular risk factors according to current American Heart Association (AHA) guideline recommendations [8, 13, 14]. Surprisingly, although the epidemiology, etiologies, mechanisms, classification, and prognostic outcomes of IS have been investigated for several decades [15–18], safe and efficacious management practices for all patients with IS, especially when recurrent IS is seriously considered, have not been fully developed. Previously, stenting for intracranial arterial stenosis (SAMMPRIS Trial), an alternative therapy to conventional medication, was conducted [19]. Regrettably, the results have been disappointing [19]. The majority of these patients, therefore, are left without any effective treatment. Accordingly, the identification of a safe and effective therapeutic option for patients with IS is fundamentally important for neurologists and physicians.

Interestingly, our previous studies [20, 21] revealed that acute IS stimulates the mobilization of endothelial progenitor cells (EPCs) into the circulation. Additionally, our other previous studies [20, 21] revealed that an increase in the number of circulating EPCs was strongly associated with favorable clinical outcomes after IS. Furthermore, our phase I and II clinical trials previously demonstrated that intracoronary transfusion of CD34 + cells improved left ventricular function in patients with diffuse coronary artery disease (CAD) and noncandidates for coronary artery intervention [22, 23]. Intriguingly, CAD and cerebrovascular disease (CVD), which constitute the majority of the same causal etiologies of endothelial dysfunction/damage and arteriosclerosis, comprise arterial obstructive syndromes. Thus, CAD and CVD are two sides of the same coin. Accordingly, we propose that autologous EPC transplantation potentially represents an alternative therapeutic modality for patients after acute IS. In fact, we conducted a phase I clinical trial of intracarotid arterial transfusion of autologous CD34 + cells derived from the peripheral circulation for older patients with IS [24], and the results demonstrated that CD34 + cell therapy was safe and offered some benefit for these patients. Surprisingly, since our design of a phase II clinical trial of CD34 + cell therapy for acute IS in 2017, no data have been reported on the efficacy of intracarotid arterial transfusion of autologous CD34 + cells for patients with acute IS worldwide. Accordingly, the abovementioned issues [1–24] and the results of our phase I clinical trial of intracarotid arterial administration of CD34 + cells for older patients with IS [24] encouraged us to further investigate the impact of intracarotid arterial transfusion of circulatory-derived autologous CD34 + cells for patients with acute IS in a randomized, open-label, controlled phase II clinical trial.

## Materials and methods

### Study design and patient population

This phase II clinical trial, which was approved by the Taiwan Food and Drug Administration (TFDA), Republic of China (IRB No: 1109012692), and the Institutional Review Board (IRB) on Human Research at Chang Gung Memorial Hospital (IRB No: 201700116A0C502) in 2018, was performed at Kaohsiung Chang Gung Memorial Hospital (CGMH), a tertiary referral center. This study was funded by a program grant from the Ministry of Science and Technology of the Republic of China (MOST 107–2314-B-182A-056-MY3). This was a randomized, open-label, controlled phase II clinical trial (clinical trial number ISRCTN15677760) to test whether intracarotid arterial transfusion of circulatory-derived autologous CD34 + cells for patients with acute IS was safe and could effectively improve neurological function and outcomes in patients after acute IS.

With the permission of the TFDA and the IRB of CGMH, this study was designed to enroll 116 consecutive patients, i.e., 58 patients in each group who had a history of acute IS [i.e., by clinical recording and brain magnetic resonance imaging (MRI) or brain computerized tomography (CT) scan findings] and were willing to participate in the study. The enrollment period was three years and started in January 2018. Unfortunately, during the enrollment period, the public health emergency resulting from the COVID-19 pandemic caused great challenges and severely affected the enrollment of the study patients. Thus, between January 2018 and March 2022, i.e., by the end of the study period, we enrolled only 28 patients (14 in the cell-treated group and 14 in the control group) who were willing to participate in the study. Since then, the clinical trial has stopped because of (1) the time needed for patient enrollment and (2) the end of financial support.

### Calculation of sample size for specific objectives

Within the study period, there were no published papers on the utilization of CD34 + cells for the treatment of acute IS. Therefore, we referred to our previous report [21] on the therapeutic effect of erythropoietin (EPO) to estimate the National Institute of Health Stroke Scale (NIHSS) scores of both the experimental and control groups for comparison. The score difference was assumed to be 2.5, and the SD was estimated to be 4.5. Based on an α = 0.05 and power = 80%, the number of participants in a group should be at least 51. With a dropout rate was 10%, 58 participants should be enrolled in each group. Accordingly, there should be 116 participants collectively in both groups. Thus, for the primary objective of the study, an estimated sample size of 58 study subjects in each group was selected on the basis of the effective size, with α = 0.05, a power of 80%, and an improvement of 1.5 in the NIHSS score in the control group vs. an improvement of 4.0 in the NIHSS score in the CD34 + cell treated group, and the standard deviation (SD) was 4.5.

### Randomization method

A total of 116 sealed envelopes were prepared for randomization. The envelopes contained a paper to assign 58 patients to receive CD34 + cell treatment (i.e., cell-treated group, i.e., 3.0 × 10^7^ CD34 + cells/patient from intracarotid arterial administration). Fifty-eight patients served as the control group, i.e., with a 1:1 homogenous distribution. The 116 sealed envelopes were then placed in a box after thorough mixing. The sealed envelopes were randomized one by one for each participant.

### Inclusion criteria

1. Age more than 45 years and less than 80 years.
2. Acute IS (onset within 14 ± 7 days, NIHSS score ≥ 8 to < 22), no midline shift or hemorrhagic transformation.
3. The patients had already received the most appropriate medical treatment, including antiplatelet treatment, blood lipid-lowering drugs, and blood pressure control.
4. The subjects/legal guardians read and understood the proposal and study purpose in detail and signed the informed consent form.

### Exclusion criteria

1. Age less than 45 years old or older than 80 years.
2. The patients had received tissue plasminogen activator (tPA) therapy or mechanical thrombectomy.
3. MRI of the brain stem and other non-middle cerebral artery-related acute ISs, hemorrhagic strokes, or the brain revealed acute ISs with hemorrhagic transformation.
4. The NIHSS score improved by more than 4 points within 12–24 h after acute IS.
5. The presence of infectious agents, such as HIV, COVID-19, AIDS or syphilis.
6. Myocardial infarction occurred within 3 months.
7. Severe stenosis of the extracranial carotid arteries (including the common carotid artery and internal carotid artery) was observed.
8. Subjects with a New York Heart Association Functional Class 3–4.
9. Malignant tumors, hematopoietic disorders, or other serious organ diseases with an expected survival of less than one year.
10. Patients with chronic kidney disease and a creatine clearance rate (CCr) < 20 ml/min were included.
11. Those who had participated in other clinical trials.
12. Patients who were not suitable for the examinations and treatments included in the present study, such as MRI.
13. Patients had other brain diseases, including tumors, infectious diseases, and neurodegenerative diseases.
14. Contraindications to G-CSF therapy.

### *Methodology for the preparation of autologous CD34* + *cells and intracarotid arterial transfusion*

The procedure and protocol for the preparation of circulatory CD34 + cells were described in our previous clinical trials [22, 23]. Specifically, prior to the isolation of peripheral blood-derived autologous CD34 + cells, granulocyte colony-stimulating factor (G-CSF) (5 μg/kg every 12 h for 8 doses) was subcutaneously administered to all participants in cell-treated group to augment the circulation of CD34 + cells (i.e., migration from the bone marrow to the circulation) for subsequent isolation via leukapheresis. After administration of the final dose of G-CSF, peripheral blood-derived mononuclear cells (PBMNCs) were isolated during leukapheresis and enriched for CD34 + cells using a commercially available device [COBE Spectra 6.1 (Terumo BCT, INC.)] at 8:00 a.m. via a double-lumen catheter inserted into the right femoral vein. These PBMNCs were harvested into a collection bag, and the tubing was chased with plasma. This cycle was repeated until the leukapheresis procedure was complete. For collection of the COBE spectra, the autoPBSC program was used with a 3–5 mL harvest volume and a 3–4 mL plasma chase. The target whole blood flow rate (WBFR) was 40–65 mL/min according to the patient’s total blood volume. The target volumes processed were approximately 10–12 L. The target collection volume was 60–100 mL. After a time interval of 4 h, abundant circulation-derived mixed population cells, predominantly consisting of autologous CD34 + cells, were isolated and ready for intracarotid arterial injection.

Generally, the COBE Spectra system uses centrifugal technology to separate whole blood into its major components. The system draws whole blood from a patient, adds an anticoagulant, separates the blood components, collects or removes specific components and returns uncollected components to the patient.

On the basis of the International Society of Hematotherapy and Grafting Engineering (ISHAGE) Guidelines for CD34 + cell determination by flow cytometry to quantify CD34 + cells in circulation, hematological stem cells were characterized by the presence of the surface markers CD34^high^/CD45^dim^/SSC^low^, which were utilized to quantify the number of isolated CD34 + cells. The formula for estimating the number of peripheral blood-derived CD34 + cells was as follows: number of CD34 + cells = (percentage of CD34 + cells) × WBC count × 10^3^ × peripheral blood stem cell (PBSC) volume (mL). In the present study, flow cytometric analysis was performed according to the current guidelines of the College of American Pathology, with a performance coefficient of variation (CV) < 4.0% (3.4 ± 2.5) (by definition, < 10.0% was considered acceptable).

Total number of CD34 + cells in the collected plasma volume always much greater than that of the 3.0 × 10^7^ CD34 + cells. By using the formula we, therefore, could exactly calculate how many plasma volume that contained 3.0 × 10^7^ CD34 + cells should be transfused into the patient. The remainder of plasma volume was immediately transferred to laboratory for cell culture and assessment of angiogenesis, flow cytometric analysis for identification circulating levels of EPCs. Definitely, 3.0 × 10^7^ CD34 + cells were transfused into one patient whereas the transfused plasma volume to each patient was various that depended on the concentration of CD34 + cells in the isolated plasma volume.

After CD34 + cells were collected, each patient was quickly transferred to the cardiac catheterization room to receive an ipsilateral intracarotid artery injection of CD34 + cells through the guiding catheter. The carotid arterial approach for the slow transfusion (i.e., for approximately 5.0 to 7.0 min) of CD34 + cells has been described in detail in our previous report [24]. Additionally, puncture of the right internal femoral vein and entry of Swan Ganz into the right internal jugular vein (RIJV) were conducted to estimate the time courses of EPCs in the RIJV.

### Flow cytometric analysis of EPC levels in the circulation and RIJV

The methodologies have been described in our previous studies [22, 23]. Specifically, the EPC populations in circulation and in the RIJV were estimated using flow cytometry with double staining, as described in our recent reports [22, 23], and a fluorescence-activated cell sorter (FACSCalibur™ system; Beckman Coulter, Inc., Brea, CA, USA). Each analysis included 300,000 cells per sample. The assays for circulatory and RIJV EPC populations in each blood sample were conducted in duplicate, and the mean levels were obtained.

Blood samples from the cell-treated group were collected at 8:00 a.m. prior to G-CSF administration, and blood samples from the other groups were collected following G-CSF treatment for flow cytometric analysis. Additionally, to assess the serial changes in EPC levels in the RIJV, serial blood samples were drawn from the RIJV at 0 min prior to and at 5, 10, and 30 min after CD34 + cell administration into the carotid artery.

### *Collection of peripheral blood-derived mononuclear cells for culturing EPCs and assessment of angiogenesis *via* the Matrigel assay*

The Matrigel assay protocol for determining angiogenesis has been described in detail in our previous reports [22, 23]. Briefly, to evaluate the angiogenesis capacity of EPCs, 10 mL of peripheral blood was collected from each patient in the cell-treated group prior to G-CSF therapy. Blood samples from each patient in the control group were also obtained at the same time points to serve as controls. After completing G-CSF treatment, 10 mL of peripheral blood was again drawn from each patient in the cell-treated group. In addition, 10 mL of plasma containing CD34 + cells that were ready for transfusion after G-CSF treatment in cell-treated patients was collected. All the samples were cultured for 21 days to assess the angiogenesis capacity of the EPCs. Flow cytometric analysis was performed to determine the cellular characteristics (i.e., EPC surface markers) with appropriate antibodies after 21 days of culture.

### *Time courses of neurological functional analysis and neuropsychological testing prior to and 6 months after CD34* + *cell therapy*

The NIHSS score, modified Rankin scale score and Barthel index were measured by a senior neurologist who was completely blinded to the patient groupings and the treatment protocol. Additionally, a clinical psychologist who was also blinded to each patient’s status conducted a neuropsychological battery of tests that focused on attention, executive function, speech, language, memory, and visuospatial functional integrity. Attention functions were measured using the digit span score from the Wechsler Adult Intelligence Scale-III (WAIS-III) [25, 26] and by the attention and orientation scores from the Cognitive Ability Screening Instrument (CASI) [25, 26]. Executive functions were assessed via digit symbol coding, similarity, arithmetic, picture arrangement, and matrix reasoning scores from the WAIS-III and abstract thinking scores from the CASI. Memory functions were measured via short- and long-term memory scores from the CASI and information scores from the WAIS-III. Speech and language abilities were assessed via vocabulary and comprehension scores from the WAIS-III and language scores from the CASI. Visuospatial functions were assessed via picture completion and block design scores from the WAIS-III and a drawing score from the CASI.

### *Definitions of the procedural success, safety and complications of CD34* + *cell therapy*

Procedural success and safety were defined as the successful administration of CD34 + cells into the cerebral circulation from the ipsilateral common carotid artery of the IS using a guiding catheter under the guidance of a fluoroscope and the absence of any complications after the procedure.

Additionally, procedure-related complications were defined as (1) vascular assessment complications or (2) new ischemic stroke/hemorrhagic bleeding after the procedure. If neurological complications were clinically suspected, a brain magnetic resonance imaging (MRI) was quickly conducted.

### *A trend of 0.05* ≤ *p* < *0.2 was defined as an improved response after CD34* + *cell therapy in the present study*

In the present study, a trend of 0.05 ≤ p < 0.2 was considered an improved response after CD34 + T-cell therapy based on our previous report [24]. Briefly, in view of the small sample size (i.e., n = 14 in each group), which could distort the statistical significance, variables with a trend of 0.05 ≤ p < 0.2 were defined as having an improved response to CD34 + cell therapy.

### Definition of angiogenesis in the cerebral circulation

In this study, an increase in the mean uptake of Tc-99 m ECD by brain perfusion SPECT was considered an increase in cerebral circulation.

### Standardized formality of the clinical follow-up by TFDA request and long-term follow-up

In addition to the regular follow-up of each patient at our outpatient clinic, a case report form that recorded all the clinical information of the patient, including the presence or absence of acute or subacute events, was designed and completed by a research nurse regularly after each visit and readmission as well as through telephone interviews on an irregular basis. The TFDA and IRB committees required that all participants be followed up for one year.

After one year of follow-up by the TFDA and IRB committees, all patients were followed up regularly at outpatient departments until the end of the study period.

### Statistical analysis

All values are expressed as the means ± SDs, numbers, or percentages, as appropriate. Continuous data were analyzed via independent or paired t tests, whereas categorical data were analyzed via chi-square tests or Fisher’s exact tests when appropriate. The time course of the variables for longitudinal follow-up was determined via repeated-measures ANOVA. The mean uptake levels of Tc-99 m ECD before and after treatment obtained using brain perfusion SPECT were analyzed with the Wilcoxon signed-rank test for paired data. Statistical analysis was performed via SPSS statistical software for Windows version 13 (SPSS for Windows, version 28; SPSS, IL, USA). A trend of 0.05 ≤ p < 0.2 was defined as an improved response after CD34 + cell therapy in the present study.

## Results

### ***Baseline characteristics of the two groups of patients (***Table 1***)***

**Table 1:** The baseline characteristics of study and control groups

Variables	Cell-treated group■ (n = 14)	Control group (n = 14)	*p* value§
Age (yrs.)	65.2 ± 8.2	64.5 ± 9.6	0.833
Sex (male) (%)	57.1% (8/14)	64.3% (9/14)	0.699
Hypertension (%)	78.6% (11/14)	85.7% (12/14)	1.0
Hyperlipidemia (%)	71.4% (10/14)	57.1% (8/14)	0.430
Diabetes mellitus (%)	35.7% (5/14)	28.6% (4/14)	1.0
Smoking (%)	14.3% (2/14)	35.7% (5/14)	0.385
Old stroke (%)	21.4% (3/14)	7.1% (1/14)	0.596
Old myocardial infarction (%)	0% (0)	7.0% (1/14)	1.0
History of coronary artery disease (%)*	35.7% (5/14)	14.3% (2/14)	0.385
Clinical presentation of heart failure (%)	0% (0)	0% (0)	–
Atrial fibrillation (%)	35.7% (5/14)	28.6% (4/14)	1.0
Body heigh (BH) (cm)	164.3 ± 7.8	163.0 ± 7.8	0.654
Body mass (BM) index	27.0 ± 4.4	26.3 ± 5.7	0.724
Creatinine (mg/dL)	0.81 ± 0.24	0.87 ± 0.61	0.713
creatinine clearance rate (CCr) (mm/min)	89.3 ± 24.2	95.7 ± 44.5	0.679
Medications upon presentation			
Aspirin (%)	85.7% (12/14)	92.9% (13/14)	1.0
Clopidogrel (%)	7.0% (1/14)	28.6% (4/14)	0.326
Statins (%)	35.7% (5/14)	57.1% (8/14)	0.256
New oral anticoagulant (%)	28.6% (4/14)	28.6% (4/14)	1.0
ACEI/ARB (%)	35.7% (5/14)	28.8% (4/14)	1.0
Calcium channel blocker (%)	21.4% (3/14)	71.4% (10/14)	0.008
Beta blocker (%)	14.3% (2/14)	28.8% (4/14)	0.648
Carotid doppler findings			
Peak systolic velocity of LCCA	76.0 ± 28.8	79.9 ± 20.9	0.688
Peak systolic velocity of LICA	69.1 ± 32.2	63.9 ± 10.0	0.572
Left carotid index†	0.88 ± 0.47	0.85 ± 0.26	0.855
Peak systolic velocity of RCCA	76.3 ± 22.4	81.3 ± 30.0	0.622
Peak systolic velocity of RICA	74.7 ± 49.6	72.7 ± 39.4	0.907
Right carotid index‡	0.96 ± 0.44	0.90 ± 0.29	0.673

*ACEIs/ARBs* angiotensin converting enzyme inhibitors/angiotensin II type 1 receptor blockers; *LCCA* left common carotid artery; *LICA* left internal carotid artery; *RCCA* right common carotid artery; *RICA* right internal carotid artery

■ Indicates patients were treated with CD34 + cells

^*^Defined as history of coronary artery obstruction > 50.0%

^†^Defined as the ratio of the peak systolic velocity of the LICA to the LCCA

^‡^Defined as the ratio of the peak systolic velocity of the RICA to the RCCA

^§^Indicates continuous data were analyzed using the independent t test, whereas categorical data were analyzed using the chi test or Fisher’s exact test when appropriate

Age and sex did not differ between the two groups. Additionally, the incidences of cerebrovascular risk factors, including diabetes mellitus and hyperlipidemia, were similar between these two groups of patients. Furthermore, the incidences of cerebrovascular risk factors, including hypertension and smoking, were also similar between the control group and the cell-treated group.

The incidence rates of previous stroke and atrial fibrillation, history of CAD, clinical presentation of heart failure and previous myocardial infarction did not differ between the two groups of patients. Additionally, the body mass index, body weight, and circulatory levels of creatinine and CCr were similar between the two groups of patients.

Except for calcium-channel blocker agent use, which was significantly greater in the control group than in the cell-treated group, the percentages of patients who used antiplatelet agents, statins, beta-blockers, ACEIs/ARBs and new oral anticoagulant agents did not differ between the two groups of patients.

### ***Circulatory hematologic findings (***Table 2***)***

**Table 2:** Circulatory hematologic findings

Variables	Cell-treated group (n = 14)	Control group (n = 14)	*p* value‡
Baseline prior to G-CSF treatment			
Red blood cells (RBC) (× 10^6^)	4.98 ± 0.60	4.49 ± 0.56	0.036
White blood cells (WBC) (× 10^3^)	8.7 ± 2.2	8.9 ± 3.8	0.866
Hemoglobin (g/dL)	14.6 ± 1.4	13.3 ± 1.7	0.033
Platelet (× 103/µL)	237.6 ± 52.3	270.9 ± 134.9	0.398
Segment (%)	72.8 ± 9.4	69.9 ± 8.3	0.395
Lymphocyte (%)	18.9 ± 8.4	19.6 ± 6.6	0.804
Compared prior to & after G-CSF treated*	Baseline†	After G-CSF treated†	
Red blood cells (× 10^6^)	4.98 ± 0.60	4.58 ± 0.57	< 0.001
White blood cells (× 10^3^)	8.7 ± 2.2	49.6 ± 12.1	0.005
Hemoglobin (g/dL)	14.6 ± 1.4	13.5 ± 1.2	< 0.001
Platelet (× 10^3^/µL)	237.6 ± 52.3	228.4 ± 67.9	0.068
Segment (SEG) (%)	72.8 ± 9.4	81.8 ± 4.4	0.408
Lymphocyte (LYM) (%)	18.9 ± 8.4	7.0 ± 2.7	0.553
HPCs (× 10^3^/µL)	–	92.8 ± 208.4	–

^*^Indicated granulocyte colony stimulating factor (G-CSF) was only administered to the cell-treated group by subcutaneous administration (5 μg/kg twice a day for 4 days)

^†^Indicated the parameters of prior to and after G-CSF treatment in group 1 patients

*HPCs* hematopoietic progenitor cells

^‡^Indicated by independent t test or t test for paired data

Prior to G-CSF treatment, the circulatory levels of red blood cells (RBCs) and hemoglobin were significantly greater in the cell-treated group compared with the control group, whereas the white blood cell (WBC) and platelet counts and the percentages of segments and lymphocytes did not differ between these two groups of patients.

Compared with the parameters prior to G-CSF treatment, among the cell-treated patients, the WBC count significantly increased, whereas the RBC count and hemoglobin significantly decreased after G-CSF treatment. Additionally, the number of hematopoietic progenitor cells (HPCs) was substantially greater in the cell-treated group than in the control group after G-CSF treatment. However, the platelet counts and percentages of segments and lymphocytes did not differ before and after G-CSF treatment among the control group patients.

CD34 + cells were successfully transferred into the cerebral circulation using the ipsilateral common carotid artery of the IS of each study patient with a catheter guided by a fluoroscope in the cardiac catheterization room. No procedural-related complications were observed in the present study during or after the procedure.

### ***Circulatory EPC levels in cell-treated group and control group patients before and after G-CSF treatment (***Fig. 1***)***

**Fig. 1:**
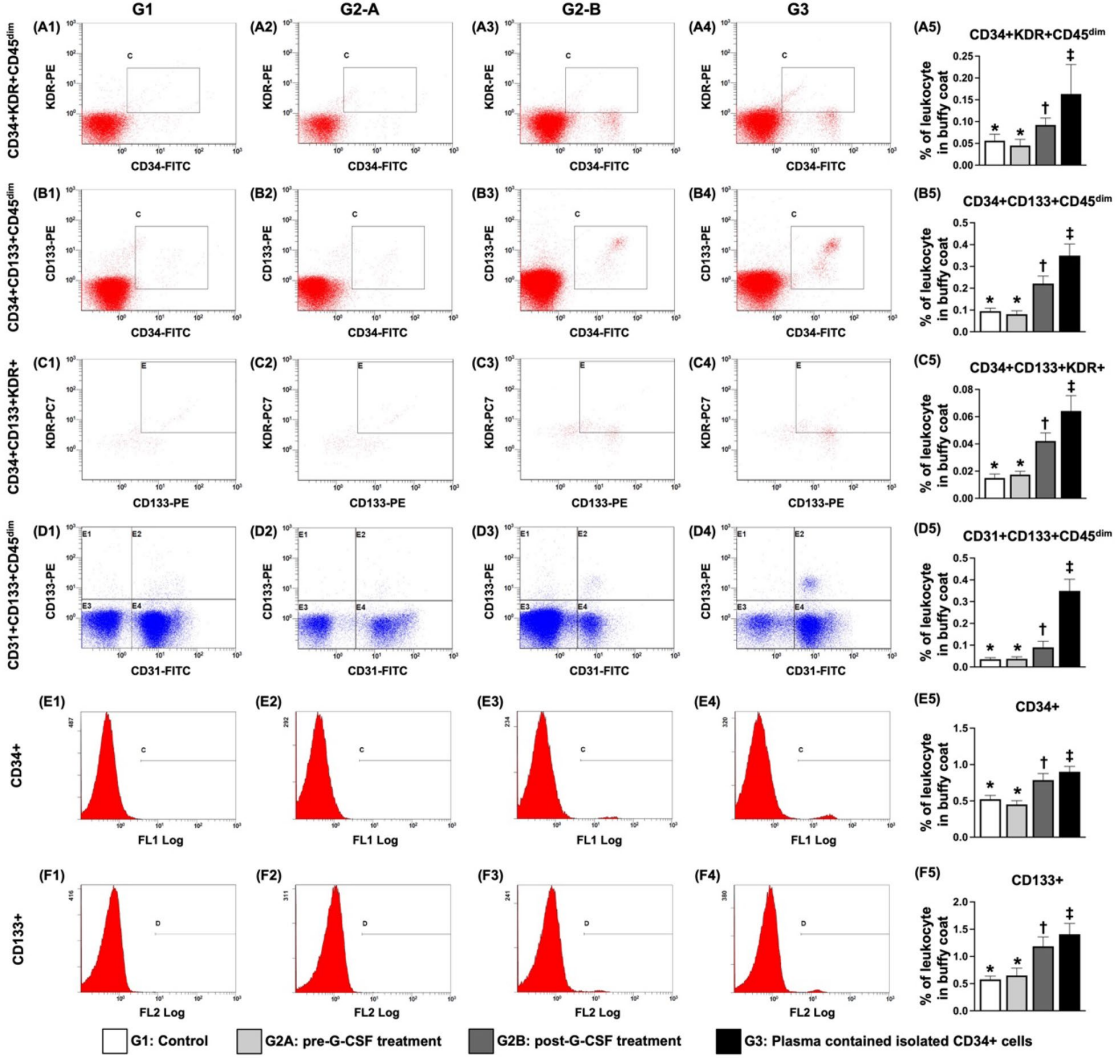
Circulatory EPC levels in the cell-treated group and control group after G-CSF treatment A1–A4 Flow cytometric analysis for the identification of CD34 + KDR + CD45^dim^ cells. A5 Analytical result of the number of CD34 + KDR + CD45^dim^ cells, * vs. other groups with different symbols (†, ‡), *p* < 0.0001. B1–B4 Flow cytometric analysis for the identification of CD34 + CD133 + CD45^dim^ cells. B5 Analytical result of the number of CD34 + CD133 + CD45^dim^ cells, * vs. other groups with different symbols (†, ‡), *p* < 0.0001. C1–C4 Flow cytometric analysis for the identification of CD31 + CD133 + CD45^dim^ cells. C5 Analytical result of the number of CD31 + CD133 + CD45^dim^ cells, * vs. other groups with different symbols (†, ‡), *p* < 0.0001. D1–D4 Flow cytometric analysis for the identification of CD34 + CD133 + KDR + cells. D5 Analytical result of the number of CD34 + CD133 + KDR + cells, * vs. other groups with different symbols (†, ‡), *p* < 0.0001. E1–E4 Flow cytometric analysis for the identification of CD133 + cells. E5 Analytical result of the number of CD133 + cells, * vs. other groups with different symbols (†, ‡), *p* < 0.0001. F1–F4 Flow cytometric analysis for the identification of CD34 + cells. F5 Analytical result of the number of CD34 + cells, * vs. other groups with different symbols (†, ‡), *p* < 0.0001. All the statistical analyses were performed via one-way ANOVA, followed by the Bonferroni multiple comparison post hoc test (n = 14 for each group). Symbols (*, †, ‡) indicate significance (at the 0.05 level). EPC = endothelial progenitor cell; G-CSF = granulocyte colony-stimulating factor. G1 = the control group; G2A (cell-treated group) = pre-G-CSF treatment; G2B = post-G-CSF treatment. G3 = plasma containing isolated CD34 + cells

First, to elucidate the time points at which EPCs were expressed, flow cytometric analysis was performed in the present study. As expected, prior to G-CSF treatment, the percentages of circulating EPCs (i.e., CD34 + KDR + CD45^dim^, CD34 + CD133 + CD45^dim^, CD31 + CD133 + CD45^dim^, CD34 + CD133 + KDR + and CD133 + and CD34 +) did not differ between the cell-treated group and the control group. However, these parameters were significantly greater in cell-treated patients after receiving G-CSF treatment and were significantly greater in patients whose plasma contained isolated EPCs. Our findings suggested that G-CSF treatment promoted the mobilization of EPCs and HPCs from the bone marrow to the circulation.

### ***Estimation of angiogenesis ***via*** the Matrigel assay in both patient groups (***Fig. 2***)***

**Fig. 2:**
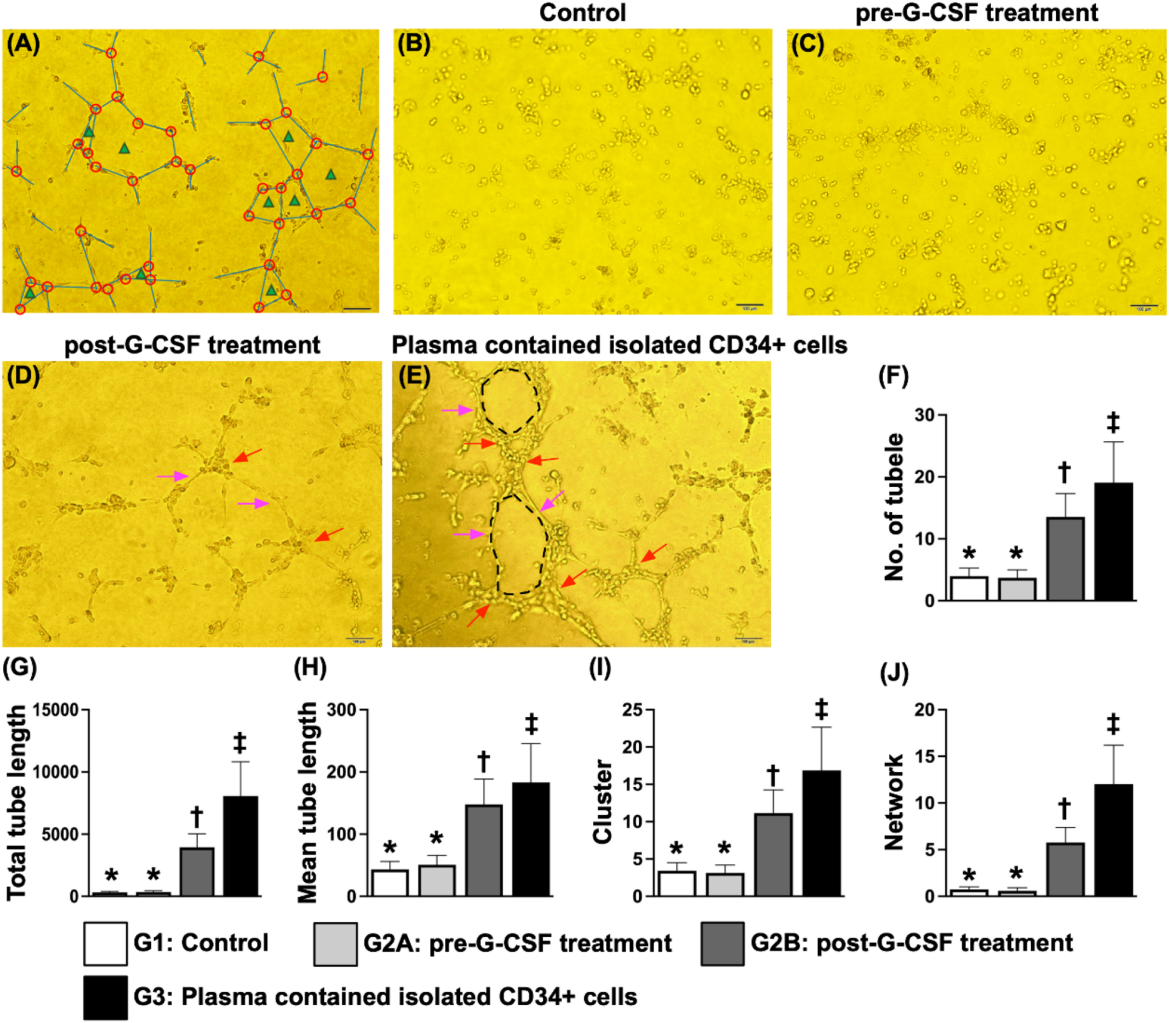
Estimation of angiogenesis using the Matrigel assay with and without G-CSF treatment in both groups of patients **A** An example of how to identify angiogenesis variables, including tubular length (blue line), cluster formation (red circle) and network formation (blue triangle). **B**–**E** Matrigel assay for identification of angiogenesis capacity in G1 (i.e., the control group) **B** and G2 (the cell-treated group) at the time points of pre-G-CSF treatment **C** and post-G-CSF treatment **D** as well as at the time of plasma isolation from CD34 + cells **E**. The pink arrows indicate tubular length. Red arrows indicate cluster formation. The black dotted line circle indicates network formation. **F** Analytical result of the number of tubules, * vs. other groups with different symbols (†, ‡), *p* < 0.001. **G** Analytical result of total tubular length, * vs. other groups with different symbols (†, ‡), *p* < 0.001. **H** Analytical result of the mean tubular length, * vs. other groups with different symbols (†, ‡), *p* < 0.0001. I Analytical result of cluster formation, * vs. other groups with different symbols (†, ‡), *p* < 0.0001. J Analytical result of network formation, * vs. other groups with different symbols (†, ‡), *p* < 0.0001. All the statistical analyses were performed using one-way ANOVA, followed by the Bonferroni multiple comparison post hoc test (n = 14 for each group). Symbols (*, †, ‡) indicate significance (at the 0.05 level). G-CSF = granulocyte colony-stimulating factor; G1 = the control group. G2A (cell-treated group) = pre-G-CSF treatment; G2B = post-G-CSF treatment. G3 = plasma containing isolated CD34 + cells

Next, we utilized a Matrigel assay to assess angiogenesis (i.e., parameters including cluster formation, tubular length, and network formation). The results revealed that these parameters were similar between the cell-treated group and the control group prior to G-CSF treatment. However, these angiogenesis parameters were significantly augmented in cell-treated patients after they received G-CSF treatment compared with both groups of patients prior to G-CSF treatment, suggesting that G-CSF enhances the angiogenesis capacity of EPCs. One particularly interesting finding was that the parameters of angiogenesis were highest in plasma containing isolated EPCs.

### ***Serial changes in the RIJV levels of stromal cell-derived factor (SDF)-1α and EPC/HPCs in 14 study patients (***Fig. 3***)***

**Fig. 3:**
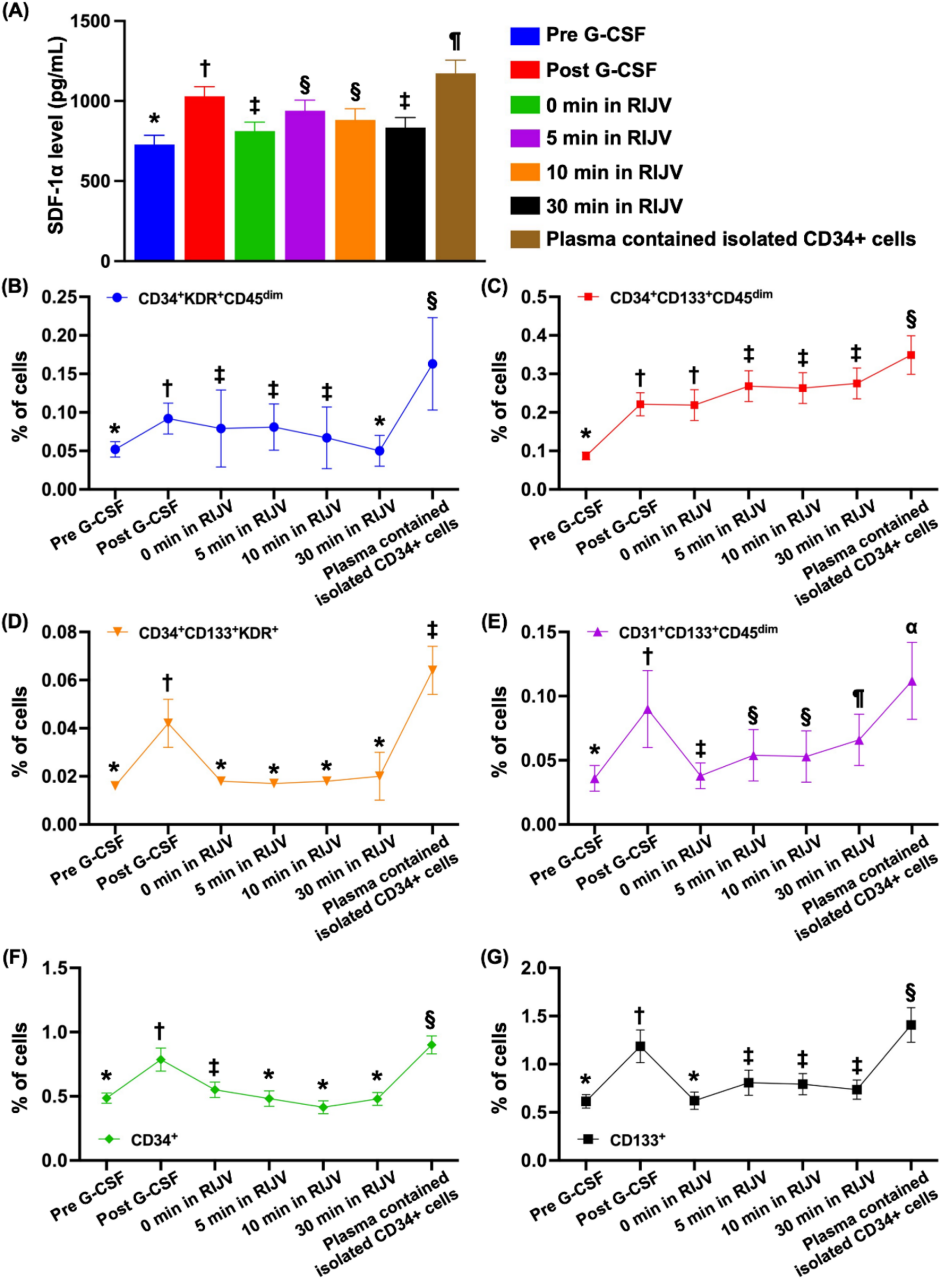
Serial changes in SDF-1α levels and the EPC/HPC ratio in the RIJV in14 cell-treated patients** A** ELISA findings for identification of the time courses of stromal cell-derived factor (SDF)-1α in cell-treated patients; analytical result of SDF-1α, * vs. other groups with different symbols (†, ‡, §, ¶), *p* < 0.0001.** B** Flow cytometric analysis of the levels of CD34 + KDR + CD45^dim^ cells at baseline and over time; analytical result of the number of CD34 + KDR + CD45^dim^ cells; * vs. other groups with different symbols (†, ‡, §), *p* < 0.0001. **C** Flow cytometric analysis of the levels CD34 + CD133 + CD45^dim^ cells at baseline and over time; analytical result of the number of CD34 + CD133 + CD45^dim^ cells; * vs. other groups with different symbols (†, ‡, §), *p* < 0.0001. **D** Flow cytometric analysis of CD34 + CD133 + KDR + cells at baseline and over time; analytical result of the number of CD34 + CD133 + KDR + cells; * vs. other groups with different symbols (†, ‡), *p* < 0.0001.** E** Flow cytometric analysis of CD31 + CD133 + CD45^dim^ cells at baseline and over time; analytical results of the number of CD31 + CD133 + CD45^dim^ cells; * vs. other groups with different symbols (†, ‡, §, ¶, α), *p* < 0.0001. **F **Flow cytometric analysis of CD34 + cells and the analytical results of the number of CD34 + cells at baseline and over time; * vs. other groups with different symbols (†, ‡, §), *p* < 0.0001. **G** Flow cytometric analysis of CD133 + cells and the analytical results of the number of CD133 + cells at baseline and over time; * vs. other groups with different symbols (†, ‡, §), *p* < 0.0001. All the statistical analyses were performed using one-way ANOVA, followed by the Bonferroni multiple comparison post hoc test (n = 14 for each group). Symbols (*, †, ‡) indicate significance (at the 0.05 level). G-CSF = granulocyte-colony stimulating factor; RIJV = right internal jugular vein; EPCs = endothelial progenitor cells

To evaluate SDF-1α levels in cerebral circulation at different time intervals after CD34 + intracarotid arterial transfusion, we collected blood samples from the RIJV and measured the level of this soluble angiogenesis factor at baseline (i.e., 0 min) and at 5, 10, and 30 min after CD34 + cell transfusion. As expected, SDF-1α levels in the RIJV at baseline (i.e., at 0 min) and at 30 min were significantly greater and further significantly greater at 5 and 10 min than the circulatory levels prior to G-CSF treatment, suggesting that EPCs, such as CXCR4 + cells, become trapped in cerebral blood vessels and capillary networks. Additionally, the SDF-1α level was significantly greater in the circulation immediately after completing G-CSF treatment and was significantly greater in plasma containing concentrated isolated CD34 + cells compared with the RIJV at 5, 10, and 30 min post-CD34 + cell treatment.

To elucidate the shedding rate of EPCs from the RIJV after intracarotid artery administration of CD34 + cells, time courses of EPC and HPC evaluations were conducted using flow cytometry. The time courses revealed that EPCs (i.e., CD34 + KDR + CD45^dim^, CD34 + CD133 + CD45^dim^, CD31 + CD133 + CD45^dim^, CD34 + CD133 + KDR + , CD133 + and CD34 + surface markers) were continuously excreted from the RIJV to the circulation at 5, 10, and 30 min after intracarotid arterial transfusion of CD34 + cells. Additionally, except for CD34 + cells and CD34 + CD133 + KDR + cells, these parameters were significantly greater at these three time points than they were prior to G-CSF treatment and at 0 min prior to CD34 + cell administration. Furthermore, among these patients, the circulating levels of EPCs were markedly greater after G-CSF treatment and were notably greater in plasma containing isolated CD34 + cells compared with before G-CSF treatment. These findings suggest that G-CSF treatment allows EPC mobilization from the bone marrow to the circulation.

### ***One-year and long-term neurological and clinical outcomes (***Table 3***)***

**Table 3:** Time courses of neurological and prognostic outcomes

Variables	Cell-treated group■ (n = 14)	Control group (n = 14)	*p* value**
Neurological disability upon presentation			
NIHSS	10.9 ± 3.2	11.9 ± 3.2	0.391
Modified Rankin scale	4.43 ± 0.65	4.36 ± 0.84	0.803
Barthel index	12.9 ± 11.0	18.9 ± 26.5	0.440
Baseline CASI score	67.1 ± 17.1	67.7 ± 25.3	0.946
Baseline WIAS-III	78.2 ± 13.2	81.8 ± 16.4	0.585
Baseline MMSE	20.7 ± 5.7	20.8 ± 7.3	0.990
Neurological disability at day 30*			
NIHSS	7.6 ± 2.5	9.8 ± 4.5	0.130
Modified Rankin scale	3.36 ± 0.75	3.54 ± 0.66	0.511
Barthel index	50.4 ± 25.2	39.6 ± 27.9	0.076
Neurological disability at day 90†			
NIHSS	6.6 ± 2.2	8.9 ± 4.7	0.144
Modified Rankin scale	3.14 ± 0.95	3.23 ± 0.83	0.801
Barthel index	59.6 ± 25.2	55.0 ± 33.8	0.688
Neurological disability at day 180‡			
NIHSS	6.1 ± 2.6	7.5 ± 4.0	0.332
Modified Rankin scale	3.0 ± 1.0	2.90 ± 0.83	0.815
Barthel index	60.0 ± 27.7	63.2 ± 29.3	0.788
Death 3 month later after acute IS	0% (0)	7.1% (1/14)	1.0
Withdrawn study 3 months after acute IS§	0% (0)	14.3% (2/14)	0.481
LAAC procedure	21.4% (3/14)	14.3% (2/14)	1.0
At 6-month CASI score	67.0 ± 21.9	65.4 ± 23.6	0.890
At 6-month WIAS-III	80.5 ± 18.1	72.0 ± 8.7	0.707
At 6-month MMSE	20.5 ± 6.3	16.8 ± 6.8	0.270
1-year combined end point	0% (0)	14.3% (2/14)	0.481
Recurrent stroke	0% (0)	7.1% (1/14)	1.0
Mortality	0% (0)	7.1% (1/14)	1.0
Long-term combined end point††	14.3% (2/14)	50.0% (7/14)	0.103
Recurrent stroke	7.1% (1/14)	14.3% (2/14)	1.0
Mortality	7.1% (1/14)	14.3% (2/14)	1.0
Severe disability#	0% (0)	14.3% (3/14)	0.222
Tumorigenesis at long-term follow up†† (Brain MRI study for each patient at 6 months after CD34 + cell treatment)	0% (0)	0% (0)	–

*NIHSS* National Institutes of Health Stroke Scale; *LAAC* left atrial appendage closure

■Indicates patients were treated with CD34 + cells

^*^Defined as the time point of the early recovery phase after acute ischemic stroke (IS)

^†^Defined as the time point of the late recovery phase after acute IS

^‡^Defined as the time point of the chronic phase after acute IS. Brain MRI studies are always utilized for the evaluation of recurrent stroke in patients

^§^Due to poorer condition, the patient was transferred to a long-term care center

Defined as recurrent stroke or death

^**^Indicates continuous data were analyzed using the independent t test, and categorical data were analyzed using the chi test or Fisher’s exact test when appropriate

^††^Brain MRI was regularly conducted for each patient at 6 months after acute ischemic stroke (note that a routine MRI study was not performed for our patients since the first brain MRI study). Additionally, regular clinical assessment of the patients at the outpatient department was performed with a follow-up time of 4.1 ± 1.3 years. No tumorigenesis was recorded during the long-term clinical follow-up

^#^Defined as bedridden and totally dependent on life care

At the end of the one-year follow-up, the mortality rates were 0% in the cell-treated group and 7.1% in the control group. Additionally, the incidence rates of recurrent stroke were 0% in the cell-treated group and 7.1% in the control group. Thus, the combined endpoints (defined as death or recurrent stroke) at the one-year follow-up were 0% in the cell-treated group and 14.3% in the control group.

The neurological functional parameters of the NIHSS score, modified Rankin scale score and Barthel index were similar upon presentation (Table 3). Additionally, the parameters of the Barthel index and the modified Rankin scale score on Days 60, 90 and 180 and the NIHSS score on Day 180 did not differ between patients in the cell-treated group and those in the control group. However, at Days 30 and 90 after acute IS, the NIHSS score improved (trend of 0.05 ≤ p < 0.2) in the cell-treated group compared with the control group. Conversely, the CASI, WIAS-III and MMSE scores, three indices of neuropsychological scoring, did not differ on Day 180 after acute IS among cell-treated or control patients.

The long-term follow-up time was 4.1 ± 1.3 years. Importantly, the combined cumulative endpoint (i.e., defined as recurrent stroke, mortality and severe disability) of this study during long-term follow-up was markedly greater in the control group than in the cell-treated group, suggesting that the favorable outcomes in cell-treated group are potentially due to CD34 + treatment. Additionally, no tumorigenesis was observed during either the one-year or long-term follow-up in cell-treated or control patients (Table 3).

Two months after acute IS, 3 patients in the cell-treated group and 2 patients in the control group underwent catheter-based left atrial appendage closure due to atrial fibrillation-induced embolic stroke and were uneventfully discharged from the hospital (Table 3).

## ***Value of the mean uptake readings of Tc-99 m ECD brain perfusion SPECT*** (Table 4*** and ***Fig. 5***)***

**Table 4:** Comparison of median uptake readings of Tc-99 m ECD brain perfusion SPECT study before (baseline) and after (by 6-month) CD34 + cell therapy in both groups of patients

Variables	Median (Q1, Q3)■ (baseline) in cell-treated group	Median (Q1, Q3)■ (6 month) in cell-treated group	*p*-value*	Median (Q1, Q3) (baseline) in control group	Median (Q1, Q3) (6 month) in control group	*p* value*
Basal ganglia (L)	321.5 (263.8, 411.8)	326.5 (278.2, 384.7)	0.507	346.5 (275.5, 361.5)	303.1 (278.2, 359.2)	0.477
Basal ganglia (R)	333.5 (277.2, 465.1)	376.3 (250.4, 485.4)	0.807	354.3 (264.6, 410.8)	402.8 (333.7, 458.0)	0.110
Central region (L)	311.7 (244.6, 392.2)	325.9 (299.3, 401.6)	0.753	307.3 (274.7, 328.6)	291.1 (270.3, 334.5)	0.374
Central region (R)	317.5 (258.8, 442.5)	351.7 (322.0, 446.7)	0.753	329.1 (271.9, 367.3)	367.5 (295.0, 415.9)	0.130
Cerebellum (L)	355.7 (288.8, 477.7)	392.7 (310.9, 546.6)	0.463	355.6 (302.5, 392.9)	423.6 (341.4, 544.5)	0.110
Cerebellum (R)	343.3 (258.3, 446.2)	360.7 (280.4, 479.4)	0.552	317.7 (286.4, 372.1)	391.6 (329.9, 446.9)	0.110
Cingulate & paracingulate gyri (L)	336.1 (270.1, 422.8)	353.2 (296.4, 437.2)	0.861	305.1 (267.3, 374.5)	355.2 (307.6, 410.5)	0.286
Cingulate & paracingulate gyri (R)	322.4 (248.6, 427.5)	343.6 (291.1, 417.5)	0.753	323.4 (275.3, 343.6)	353.4 (300.6, 415.3)	0.213
Frontal lobe (L)	321.9 (250.3, 396.6)	325.3 (296.5, 412.0)	0.972	326.0 (262.9, 362.1)	333.3 (294.6, 375.8)	0.182
Frontal lobe (R)	303.7 (248.8, 426.9)	333.5 (294.3, 428.1)	0.753	299.4 (227.1, 321.4)	342.4 (308.6, 412.5)	0.131
Mesial temporal lobe (L)	293.4 (228.3, 388.3)	306.5 (233.0, 360.2)	0.753	305.1 (249.0, 351.8)	284.1 (254.7, 334.8)	0.286
Mesial temporal lobe (R)	299.7 (248.0, 395.9)	324.1 (286.5, 413.9)	0.807	357.8 (331.5, 391.8)	360.8 (289.0, 384.6)	0.131
Occipital lobe (L)	372.0 (307.9, 485.0)	410.7 (326.4, 486.1)	0.701	363.5 (304.8, 422.2)	379.5 (323.4, 476.3)	0.182
Occipital lobe (R)	362.6 (300.1, 504.0)	415.9 (372.8, 509.3)	0.6	320.1 (290.2, 348.9)	415.3 (328.2, 484.9)	0.110
Parietal lobe (L)	343.4 (274.0, 399.5)	346.0 (322.4, 418.2)	0.861	330.1 (281.4, 380.8)	311.2 (283.0, 385.2)	0.182
Parietal lobe (R)	333.9 (280.0, 466.9)	378.0 (330.1, 468.3)	0.701	332.4 (274.8, 369.6)	388.9 (301.3, 425.6)	0.110
Temporal lobe (L)	336.1 (272.0, 389.9)	344.9 (268.9, 412.5)	0.701	364.5 (287.2, 397.6)	315.9 (269.8, 372.2)	0.328
Temporal lobe (R)	333.8 (281.0, 477.6)	384.9 (341.7, 481.4)	0.701	378.6 (328.9, 468.7)	394.6 (324.1, 460.5)	0.110

*L* left, *R* right, *M* month, *SD* standard deviation

■ Indicates patients were treated with CD34 + cells

^*^Indicates statistical analysis via the Wilcoxon signed-rank test for paired data

To evaluate whether intracarotid arterial administration of circulatory-derived autologous CD34 + cells increased cerebral blood perfusion, Tc-99 m ECD brain perfusion SPECT was conducted, and the median value was used to determine the change between baseline and 6 months after acute IS in the cell-treated group and the control group. Surprisingly, 100% (18/18) increased Tc-99 m brain perfusion uptake in all 18 different regions of the brain was detected in cell-treated patients, whereas only 72.2% (13/18) increased Tc-99 m brain perfusion uptake in the same regions was detected in control patients (i.e., 100% vs. 72%, *p* = 0.046, as measured by Fisher’s exact test for count data), indicating that angiogenesis in the cerebral system was significantly greater in the cell-treated group than in the control group.

### ***Time courses of neurological function at the one-year follow-up (***Fig. 4***)***

**Fig. 4:**
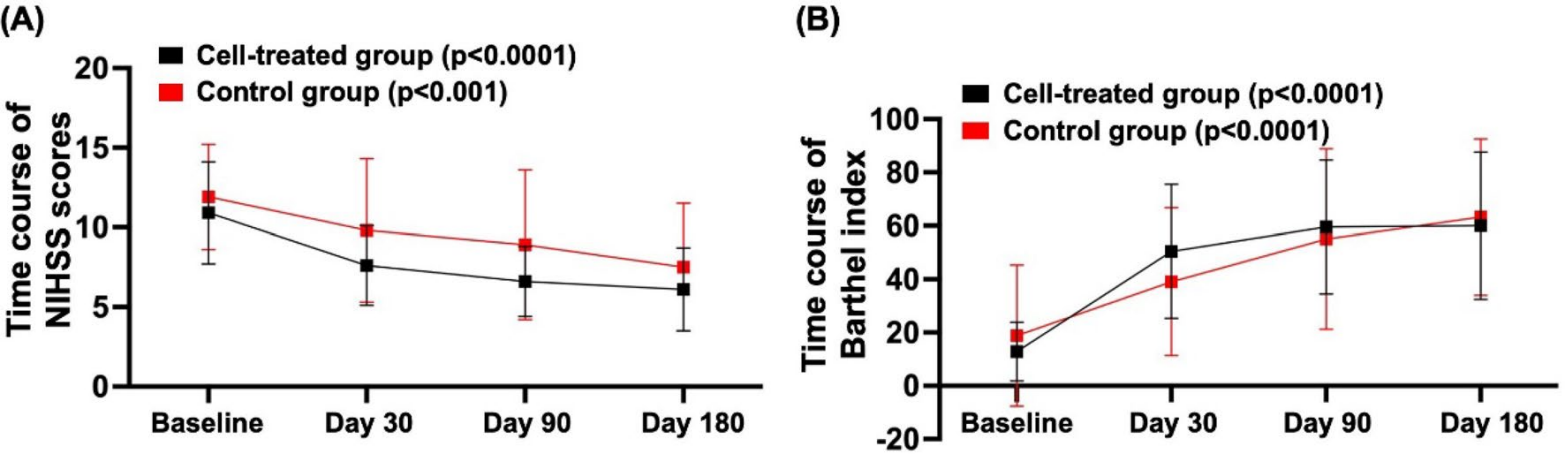
Longitudinal evaluation of serial changes in the NIHS and Barthel index in each group of patients **A** Time courses of the National Institute of Health Stroke Scale (NIHSS) scores in the cell-treated group and control group. The results were analyzed via repeated-measures ANOVA, with *p* < 0.0001 in cell-treated group and *p* < 0.001 in control group. **B** Time courses of the Barthel index in the cell-treated group and control group. The results were analyzed via repeated-measures ANOVA, with *p* < 0.0001 in the cell-treated group and *p* < 0.0001 in the control group

**Fig. 5:**
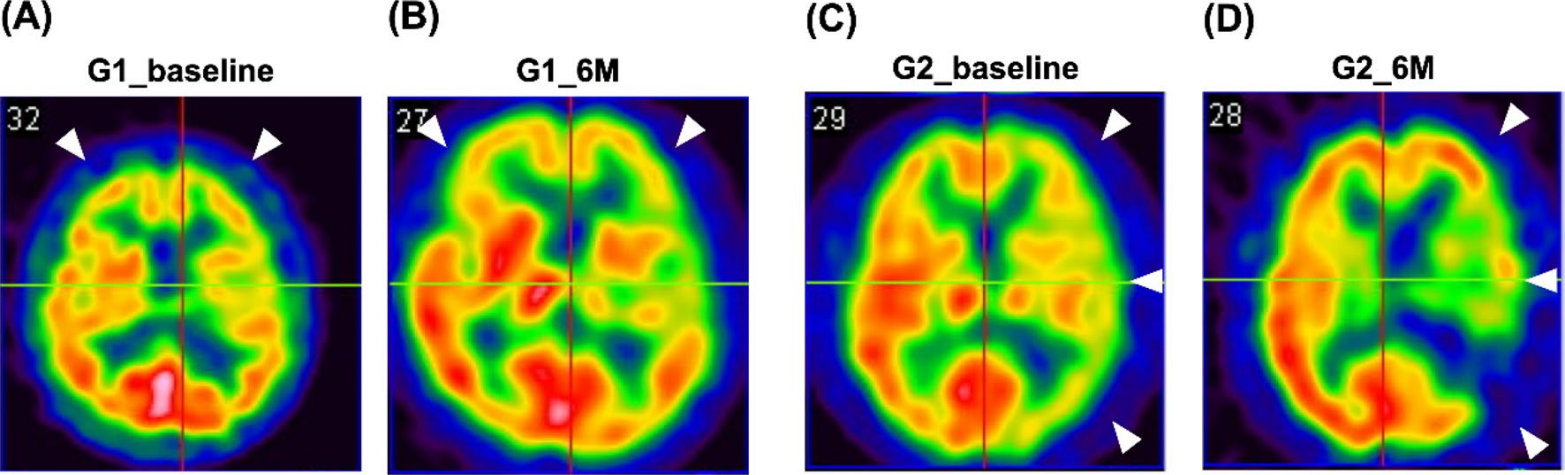
Images from brain perfusion single-photon emission computed tomography (SPECT) studies using 99mTc-labeled radiopharmaceuticals **A** and **B** Images indicating the baseline (**A**) prior to and 6 months (**B**) after CD34 + T-cell therapy in the cell-treated group (G1). **C** and **D** Baseline (**C**) and 6-month follow-up (**D**) data after acute ischemic stroke without CD34 + cell therapy in the control group (G2) (i.e., the control group). In the cell-treated group, the results of Tc-99 m ECD brain perfusion SPECT revealed that, compared with that at baseline, brain perfusion (white arrows) was notably increased by 6 months after CD34 + T-cell therapy. On the other hand, in the control group, compared with that at baseline, brain perfusion (white arrows) was markedly decreased at 6 months after acute ischemic stroke

When examining the longitudinal follow-up of prognostic neurological functions, we found that the net changes (i.e., ∆ change) in improvements in neurological function (i.e., including the NIHSS score and Barthel index) at Days 30 and 90 after IS were greater in the cell-treated group than in the control group (Fig. 5).

## Discussion

This phase II clinical trial investigating the impact of intracarotid arterial transfusion of peripheral blood-derived autologous CD34 + cells yielded substantial amounts of clinical information. First, the results demonstrated that the procedural success and safety rates were 100% and 0%, respectively, for the CD34 + cell-treated group, and no tumorigenesis was observed during long-term clinical follow-up in the study patients. Second, compared with those of the control group, the NIHSS scores greatly improved at both the early (defined as Day 30) and late (defined as Day 90) recovery phases in the cell-treated group after acute IS. Finally, the combined endpoint of long-term follow-up (i.e., mean follow-up time: 4.1 ± 1.3 years) was markedly lower in cell-treated patients than in control patients.

Although the safety and efficacy of autologous EPC/CD34 + cell therapy have been extensively investigated in the context of ischemic heart disease [22, 23, 27–30], the use of this novel strategy for the management of patients after IS has been infrequently reported [24, 31, 32], indicating that considerable scientific research is still needed. This phase II clinical trial was designed by our group based on a spatiotemporal context. One important finding in the present study was that all of the treatments administered to patients who received CD34 + cell therapy were safe, and no tumorigenesis was observed during the 1-year or long-term follow-up. Intriguingly, our previous phase I clinical trial of old patients with IS treated by autologous CD34 + cell intracarotid arterial transfusion [24] and two other previous clinical trials [31, 32], including one involving direct intracerebral implantation [31] and another involving intracranial artery injection [30] of autologous derived stem cells, have consistently revealed that cell therapy for patients with IS is safe and tolerable. Accordingly, the findings of our current and previous phase I clinical trials [24] and two other previously reported clinical trials [31, 32] support autologous CD34 + cell therapy, as no additional safety or ethical issues need to be considered.

Interestingly, two previous clinical trials [31, 32] demonstrated that hematopoietic stem cell (i.e., bone marrow-derived mononuclear cell or circulatory-derived CD34 + cell) treatment effectively improved neurological outcomes in patients after IS. However, when assessing these reports [31, 32] in detail, we found that the “efficacy” emphasized by the authors depended on only the clinical follow-up parameters and some of the imaging studies, indicating that this is still an unmet need when the underlying mechanistic basis regarding how to effectively improve clinical outcomes is considered. Compared with these previous studies [31, 32], our findings from this phase II clinical trial provided several impactful and novel findings that should be shared with the readers. First, the results of our cell culture study using the Matrigel assay demonstrated that the angiogenesis capacity was markedly increased after G-CSF treatment in cell-treated patients, indicating that this strategy not only increased the number of hematopoietic progenitor cells/CD34 + cells that mobilized into the circulation from the bone marrow nidus but also suggested that G-CSF treatment potentially restores and rejuvenates hematopoietic progenitor cells/CD34 + cells. Second, when blood samples were serially collected and flow cytometric analysis was performed, we found that, compared with baseline levels, the shedding of EPCs from the cerebral circulation to the RIJV was continuously high at 5, 10, and 30 min after intracarotid arterial transfusion of autologous CD34 + cells, suggesting that the majority of transfused CD34 + cells were still retained/trapped in the cerebral circulation for a rather long duration for potential use in angiogenesis and as sources of secretory angiogenesis factors rather than the original hypothesis that they would be completely removed from the brain to the systemic circulation within several cardiac outputs (usually 70 cardiac outputs in 60 s with a total blood volume of 5000 mL to be pushed out from the heart to the circulation). Third, ELISA of blood samples from the RIJV revealed that, compared with those from the baseline and control groups in circulation, the level of SDF-1α, a ligand of EPCs/CXCR4 + cells, was markedly increased at blood sampling times of 5, 10 and 30 min, suggesting that this angiogenesis factor plays a crucial role in trapping EPCs/CXCR4 + cells (i.e., a ligand and receptor biding together) in cerebral tissues/microvasculature, explaining why the number of shedding EPCs was persistently increased in the circulation of the RIJV (i.e., a reflection of cerebral circulation). Fourth, the degree of perfusion of different brain regions, as evaluated by Tc-99 m ECD brain perfusion SPECT (Table 4), was markedly increased in cell-treated group compared with control group, suggesting that CD34 + cell therapy may restore some blood perfusion in the cerebral circulation and microvasculature through angiogenesis/neovascularization. Our findings of increased angiogenesis and soluble angiogenesis factors in cerebral circulation after CD34 + cell transfusion could, at least in part, reveal the underlying mechanisms or biological pathways for neuroprotection and improvement of neurological function after acute IS. Interestingly, our previous experimental study [33] demonstrated that angiogenesis and the generation of angiogenesis mediators play crucial roles in improving neurological function and outcomes in rodents with acute IS treated with intracarotid transfusion of autologous EPCs. Additionally, our previous clinical trials [22, 23, 27] revealed that intracoronary transfusion of circulation-derived CD34 + cells in patients with end-stage diffuse coronary artery disease who were not candidates for coronary intervention significantly improved heart failure and heart function, mainly through angiogenesis and neovascularization. Furthermore, our previous study [21] also revealed that erythropoietin (EPO) therapy markedly improved neurological outcomes in patients after acute IS predominantly by increasing the levels of circulating EPCs. Interestingly, we previously reported that an increase in the levels of circulating EPCs was an independent predictor of favorable prognostic outcomes in patients after acute IS [20]. Thus, our previous findings [20–23, 27], in addition to being consistent with the findings of our present study, support the mechanistic basis by which CD34 + cells enhance angiogenesis to reverse neurological outcomes in patients with IS.

Finally, the primary finding in the present study was that the neurological functional parameters of the National Institutes of Health Stroke Scale (NIHSS) score, an indicator of neurological outcome, improved markedly (i.e., with a trend toward statistical significance) in the cell-treated group compared with the control group in the early and late recovery phases (Table 3). Additionally, when we carefully examined the longitudinal follow-up of serial changes in the NIHSS score and Barthel index, we found that these two parameters, especially the NIHSS score, were more notably improved in the cell-treated group than in the control group (refer to Fig. 4). Furthermore, the long-term combined endpoint was markedly lower in the cell-treated group than in the control group, indicating that the prognostic outcome was favorable in patients with acute IS who received CD34 + cell treatment. Our findings suggest that early intracarotid arterial transfusion of autologous CD34 + cells might be beneficial for patients after acute IS, especially when long-term outcomes are considered.

Growing preclinical studies have shown that either intravenous or intracarotid artery administration of CD34 + effectively reduced the brain infarct volume and preserved the neurological function in both acute IS and chronic IS animal models [34–36]. These studies raise the time points at which CD34 + cells are administered to patients after acute IS should be considered a cardinal issue. Intriguingly, our recent experimental study [33] demonstrated that compared with late administration (i.e., at 7, 14 or 28 days after IS), early administration (i.e., at 3 h or 3 days after IS) of circulation-derived EPCs was more effective at reducing the brain infarct volume and improving neurological function and molecular–cellular outcomes after acute IS in rats. Interestingly, other previous preclinical studies [37, 38] have also shown that there was a therapeutic window for intravenous administration of autologous bone marrow or human umbilical cord blood derived mononuclear cells after cerebral IS in rodents. Although the time window is limited, it is commonly wider than compared to conventional pharmacological treatment [38]. The study even found that later transplantation more than 14 days after acute IS did not show any benefit on improving outcomes [38]. In this way, the findings from previous preclinical study were [37, 38] was comparable with the findings of our recent study [33]. Additionally, previous phase I and phase II clinical trials [39, 40] have demonstrated that various types of stem cell therapy, i.e., those involving bone marrow mononuclear cells, multipotent adult progenitor cells, CD34 + stem cells, or allogeneic umbilical cord blood, could potentially improve neurological outcomes in patients after IS. Interestingly, when we examined the time interval of cell administration, we found that the time points of cell therapy varied from 1 to 9 days after ischemic stroke. The results of our study along with findings from previous studies suggest that a longer intervention window (i.e., cell therapy) is potentially present in patients after acute IS. This notion could also partially explain why the time interval of catheter-based thrombectomy, ranging from 4–6 h to 24 h after acute IS, still offers some benefit for acute IS patients.

Interestingly, when we reviewed our previous clinical studies, we also reported that early administration, especially combined with repeated administration, was superior to late administration of MSCs for protecting major organs against (1) acute respiratory distress syndrome (ARDS)-induced lung damage [41, 42], (2) diabetic kidney disease-induced deterioration of residual renal function [43], and (3) brain death-induced remote organ damage and acute rejection after heart transplantation [43]. The results from our preclinical studies suggest that early and repeated doses of stem cell therapy exhibit therapeutic potential for patients with different disease conditions that are refractory to conventional therapy, and cell therapy is considered the last resort.

In the present study, we were only permitted by TFDA to administer CD34 + cells to patients with IS within 14 ± 7 days of IS onset. On the basis of our recent findings [33] and the findings of previous studies, we suggest that early administration of CD34 + cells to our patients may provide much better outcomes than that noted within 14 ± 7 days of IS onset, as employed in this clinical trial.

In conclusion, the present study used imaging studies, neurological functional and neuropsychological evaluations, clinical follow-up, and basic research studies to provide convincing scientific data to demonstrate that intracarotid arterial injection of autologous CD34 + cells was safe and potentially effective for improving favorable short- and long-term outcomes in patients after IS.

This study has several limitations. First, as mentioned in the Introduction, the small sample size did not completely comply with the designed calculation of the sample size given the occurrence of COVID-19 pandemic during the enrollment period. Second, we could not completely rule out that the statistical significance was greatly distorted due to the small sample size. Third, the NIHSS score and Barthel index did not differ at the 180-day follow-up between cell-treated group and control group. The lack of a difference could be attributed to one patient who died and another patient in control group who withdrew from the study within 3 months after acute IS in addition to the small cohort size. The two patients had relatively greater NIHSS scores and notably lower Barthel index scores. Thus, the neurological functional parameters obtained 180 days after IS should be interpreted with caution. Finally, considering the small sample size in the present study, we defined a p value < 0.2 as indicative of a trend of statistical significance. However, this threshold of 0.2 is still relatively high even for trends in the study. Thus, the data presented here should be interpreted with caution.

## Data Availability

The datasets used and/or analyzed during the current study are available from the corresponding author upon reasonable request.

## Abbreviations

CAD: Coronary artery disease
CCr: Creatine clearance rate
EPC: Endothelial progenitor cell
G-CSF: Granulocyte colony-stimulating factor
HPCs: Hematopoietic progenitor cells
HSCs: Hematopoietic stem cells
IS: Ischemic stroke
NIHSS: National Institute of Health Stroke Scale
RIJV: Right internal jugular vein
tPA: Tissue plasminogen activator
